# Selections and Abstracts

**Published:** 1913-11

**Authors:** 


### SELECTIONS AND ABSTRACTS

### THE PROSECUTION OF QUACKS.

The Chicago Tribune gives short shrift to medical fakers. It is one of those high-class newspapers, whose numbers are happily increasing, that refuse to pollute their pages with the advertisements of quacks. For the past two or three weeks the Tribune has had some members of its staff investigating the methods of Chicago advertising quacks of the "men's specialist" type, and in recent issues has given the results of these investigations. In showing up the villainous character of this disreputable business, this paper has done a distinct service to the public. When the first exposure was printed, the Tribune had a pertinent editorial on the subject, in which it asked the question:

"Gentlemen of the American Medical Association and of the Chicago Medical Association, what are you going to do about it?"

And closed with the statement:

"This obscene traffic should be rooted out. It should be destroyed in Chicago. It should be destroyed wherever it goes on in the country, and it goes on everywhere. This is work for the American Medical Association to push energetically and to insist on the authorities completing."

The Tribune is mistaken in assuming that it is the work or duty of the American Medical Associaton to take any part in the prosecution of quacks, whether they operate locally or nationally. As scientific organizations, neither the American Medical Association nor the Chicago Medical Society is morally or legally concerned with this problem. The duty devolves explicitly and directly on the officers of the law who have been elected by the people to protect them against fraud. Physicians individually and collectively, through the American Medical Association, have for years been exposing the fraud practiced on the public by the quacks and "patent-medicine" fakers. The medical profession, with but one or two notable exceptions, has been well-nigh alone in this work. Vast vested interests are involved in the dirty business of both quackery and nostrum exploitation. The powers that should have protected the public have not acted, either because the public itself was indifferent on the question—being ignorant of the menance—or because there was little to be gained and much to be lost by fighting firm-rooted evil. Public prosecutors have possibly hesitated to act because they knew that, in many instances, the newspapers whose support they desired were not only out of sympathy with such prosecutions, but were actually participating in the unholy profits of the quack and the nostrum vender.

To leave such prosecution to the medical profession is to invite the inevitable cry, on the part of the quack, of "persecution" by the "doctors' trust." With a shrewd lawyer—and the quacks employ just such men, the length of their purses being-well-nigh unlimited—it is not difficult to persuade the average lay jury that the plausible quack is being haled into court, not because he has defrauded the public, but because he has incurred the enmity of the reputable physicians, whom the public look upon as competitors! Time and again has this fact been brought out and the quack has been found "not guilty," the prosecution has failed and the swindler has gone forth, tongue in cheek, in the pose of a persecuted, much-maligned martyr.

When a crude swindler sells to the unsophisticated a "gold-brick," the officers of the law do not hesitate to prosecute. Yet here the pocketbook only is depleted; life and health are not involved. With the medical swindler, however, it is different, and action is seldom taken unless public officials are spurred into action by forces that should never need to be invoked. It is possible, of course, that the "gold-brick" swindler would be equally immune from prosecution if influential newspapers received large advertising contracts from people engaged in the "gold-brick" industry.

Let the officers of Chicago or of Cook County, whose duty is to protect the public, proceed against these "men's specialists" quacks, exposed by the Chicago Tribune. Let the State Board of Health revoke the licenses of such men as are employed in these "men's specialists" offices. There is no more reason for invoking the services of the American Medical Association or the Chicago Medical Society in this work than there would be for callng on the American Chemical Society to prosecute the street faker who offers to sell for a dime a wonderful mixture for turning brass or copper into silver. All that can be asked of the American Medical Association is that it should enlighten the public on the dangers of quackery. This the Association, through THE JOURNAL has done, is doing and will continue to do, and the effect of this enlightenment is beginning to be felt in a national movement toward purging the newspapers of the country of medical frauds and fakes. More than this should not be expected of the medical profession. In fact, it is an economic and social error to attempt to shift the responsibility for prosecutions of this sort from the shoulders where it rightly belongs.

—Editorial, Jour. A. M. E.

---

### MENINGITIS OF THE EPIDEMIC TYPE IN CHILDREN BELOW TWO YEARS OF AGE.*

HENRY KOPLIK, M. D., NEW YORK.

The diagnosis and treatment of cerebrospinal meningitis in children below 2 years of age has become a very important problem. Without fear of overestimating our modern advances in methods of diagnosis and treatment, it may be said that in older children, between the ages of 2 and 10 years, this dread affection has been deprived of its many terrifying aspects. The disease in these older children is easily diagnosed and the treatment now in vogue with the Flexner serum has given the disease a prognosis in epidemic periods equal to that of a lobar pneumonia, and in sporadic cases even a better prognosis than the pulmonary affections. In sets of sporadic cases the outlook

*Read before the Clinical Conference in February, 1913, Mount Sinai Hospital.

is not worse than in any ordinary affections of an infectious nature in childhood. The children and infants below 2 years of age often present symptoms which are misleading and, therefore an early diagnosis is not made in a number of cases. This would seem strange to some who, from the text-book description, expect a meningitis in a young child or infant to present certain definite symptoms. The diagnosis is often masked, the physician not even entertaining a thought of meningitis. It is the fear of making a lumbar puncture and finding a clear fluid normal in every way, with the consequent doubting attitude of those about the patient, that must deter many from performing this necessary aid to an early diagnosis. In no other way can we account for the large number of infants who are sent to the hospital quite late in the disease without any diagnosis having been made. Even in the hospital, with the young patient under our direct observation, it is often difficult to come to a conclusion before lumbar puncture. Particularly difficult are the cases in infants which are complicated with a pneumonia. Many cases of pneumonia are complicated with cerebral symptoms; there is the restlessness, the rigidity, the retraction of the head and the fever which continues a long time past the initial phase of the disease. Again, there are in young infants a number of cases in which the initial symptoms of fever and restlessness are not combined with any other symptoms, such as rigidity; or there may be a slight concomitant otitis to mislead us at first into the feeling that we are dealing with a purulent ear disease instead of a meningitis. The remittence or intermittence of a high temperature with increasing restlessness, and finally the appearance of neck-rigidity then shows us that we have had a meningitis from the outset. In fact, many diseases of infancy, even a simple intestinal disorder, are so often combined with milder forms of cerebral symptoms that we can easily explain how a meningitis in younger children and infants is often overlooked. The physician is alive to the presence of a Kernig, but this is certainly absent in many infants and is of little value in the diagnosis of a meningitis. Again, the onset in many diseases is with convulsions or repeated convulsions, and it would be rash to conclude that in every such case a meningitis might be present. An observation period is therefore assumed to be necessary and then valuable time is unavoidably lost.

Therefore, the first impression we obtain in a study of a series of cases of meningitis in the very young is that in all cases we should never lose sight of the possibility of meningitis and that perfection and accuracy in diagnosis in these subjects are essential.

I say, without fear of dispute, that a day's delay in older children is not dangerous (as will be seen from a study of the subject), but the same delay in a baby may mean its loss. The stress of diagnosis must be placed on the persistence of cerebral symptoms, high fever and, what is of greatest import and scarcely appreciated at its full value, Macewen's percussion note over the frontoparietal juncture as a sign of increasing fluid in the head. The younger the child the more difficult it will be to come to a definite conclusion as to increase of fluid in the ventricles, for in rachitic infants a Macewen percussion note is normal, but some practice will teach the difference in the line between the normal and abnormal. A tense fontanelle is of some value, but not always, for some children will mislead the most expert when a crying spell is present.

The mortality in young infants in cerebrospinal meningitis is quite high. In the statistics prepared by Flexner of his serum cases, of 125 infants below one year of age, treated by serum, 63 recovered and 62 died; a gross mortality of 50 per cent. On the other hand, 5 infants injected in the first three days of the disease recovered, and of 16 patients injected in the first week 12 recovered (75 per cent.) In other words, the earlier the injections the more certain the therapeutic effects of the serum. In another statistical report, giving an analysis of 400 cases, there is a mortality of 50 per cent. in 22 patients of 1 year and below, and 42 per cent. between 1 and 2 years of age. Taking these figures as a basis of comparison, in our own service in the Mount Sinai Hospital, there are 15 patients below one year of age with a mortality of 10, or 66 per cent. Of these 15 patients, 2 injected on the fifteenth day recovered, one injected on the seventeenth day improved (discharged), one on the twenty-first day was cured, and one on the twenty-ninth day cured. None of the remaining patients came to us before the second week of the disease and some not before the forty-second day. Not all the patients coming early recovered, for one injected as early as the third day and another on the fourth day of the disease died. Between the ages of 1 and 2 years the mortality was less. Of 12 patients, 6 recovered. Some of the patients who recovered came to us quite late and were injected the twenty-first day or the twenty-ninth day of the disease and recovered, whereas one patient coming to us and receiving the serum on the second day of the disease died after an illness of fourteen days.

There are other causes which can account for the high mortality, among which must certainly be placed the virulence of the infection. Not only are the cerebrum and its fissures most delicately and minutely constructed in these infants, but the infection differs widely in virulence. This has been remarked by Flexner. Another reason is that quite early at the outset in many infants the subarachnoid space at the base of the brain is cut off from that of the cord, with the result that lumbar puncture with introduction of serum becomes fruitless quite early—on the second or third day of the disease in some infants.

A proof that the virulence in various strains of meningococci plays a most important role and is not reached by the serum is the fact that it was our fortune in pre-serum days (in spite of a very high mortality in these infants by the methods of treatment then in vogue) occasionally to save a very young infant suffering from meningococcic meningitis by the simple method of repeated lumbar puncture. In an attempt to save the cases in which the subarachnoid space was closed off from the spinal canal and simple lumbar puncture was fruitless, and serum could not be introduced by this route, we have resorted to ventricular puncture, the ventricle being punctured on one or both sides, a certain amount of fluid withdrawn and serum introduced. Eight patients were treated in this manner; some of them had had lumbar puncture and others not. In all of these cases the method was fruitless. In some coming to us on the sixteenth day of the disease, the lumbar puncture being fruitless, no fluid obtained, ventricular puncture was made from the start with no results. This disheartened us as to the future of ventricular puncture. The infants do not seem to bear the procedure well and it seems to me that some improvement over simple ventricular puncture must be devised. Some infants below one year of age cannot recover from the first shock of the infection from meningococci, and lumbar puncture does not relieve them but seems to add to their shock, which becomes more evident when serum is introduced, for they may pass out suddenly or soon after the puncture and serum introduction.

There is a set of cases which cannot be reached by the serum. We have recently had such a case in an infant 10 weeks of age, suffering from purulent meningococcic meningitis, which passed out while the serum was being introduced, the variation in cerebral pressure being too rapid for such a very delicately constructed nervous system. I would therefore urge that, in infants below 2 years of age, in whom meningitis is suspected, an early lumbar puncture be made so that the treatment may be begun as soon as feasible. In these young patients fluids should be withdrawn slowly and with the greatest caution and the serum introduced very slowly in order that traumatism and extreme changes of pressure be avoided.

In young patients the temperature is a guide of great value as to the necessity of repeated puncture and introduction of serum as in older children, but in face of great restlessness and a normal temperature fluid should be withdrawn by lumbar puncture and examined for bacteria. If these persist and a cloudy fluid is obtained, it is safe to reintroduce serum rather than delay and wait for actual culture to determine whether or not such fluid is sterile. This brings me to a discussion of a baffling feature of this disease in infants and children below 2 years of age. I have mentioned the closure of the foramen of Magendie and the impossibility of performing lumbar puncture in many cases. We then see developed the hopeless picture of posterior basic meningitis. But more baffling are the cases of infants in whom, after repeated injections of serum have been made and the temperature having dropped to the normal and stayed there for weeks, we have developed under our eyes the various features of a progressive hydrocephalus. This occurs even when the fluid withdrawn is shown after a time to be sterile. This hydrocephalus is not only due to changes in the ependyma of the ventricles, but, as I have shown and also know, to a persistence of the meningococci in the ventricles in much diminished numbers and of reduced virulence.

I have tried in this summary of our practice in the wards to point out the great difficulties as we meet them in the diagnosis and treatment of these cases and to emphasize the necessity for caution before pronouncing such patients cured. Until some time has elapsed since the onset of normal temperatures, the possibility remains that hydrocephalus may intervene, and I would emphasize the fact that much still remains to be done before we can rest satisfied with our means of aiding these pathe street from our patient, so the possibility of infection by a carrier must be considered.

The chief interest in this case lies in the age of the patient, it being the first case I had seen or known of under two years. My impression was that such cases were extremely rare, so I determined to look the subject up.

Incidental. There is a surprising difference of opinion among authorities on this subject, Holt and others stating that typhoid is very rare under two years of age, Fischl and Prague, claiming that it is by no means uncommon. Chapin and Pisch estimate that 6 per cent. of all cases occur in infants.

Newborn, or young infants whose mothers are suffering from typhoid, may give a positive Widal reaction without other symptoms, possibly having had the disease in utero, or else the agglutinizing power may have passed to the child through the placenta or through the mother's milk. As in adults the largest number of cases occurs during the fall season.

Pathology of the disease differs considerably from the adult form, especially as regards the intestinal lesions. In infants there is congestion and very slight ulceration, oftentimes none at all of the Peyer's patches, palitory follicles. The post-motem appearances may be difficult to distinguish from that of colitis.

The constant enlargement of the spleen is quite noticeable in infants, this organ being found post-mortem to be very much smaller and quite soft.

The onset is often very sudden, with high fever, vomiting and sometimes convulsions. Occasionally, however, it may be gradual, with daily increasing rise of temperature during first week. The course is shorter on the average than the adult form and the mortality not so high, though Aut claims the reverse to be the case. Two weeks is given by several authors as the average duration of the fever, and in the very young it may last only eight days.

The fever may have the typical adult form, first the gradual rise, then the sustained high fever with regular daily remissions, followed by a gradual fall to normal, but as a general thing it is more irregular than the adult.

The tongue is often characteristic, having a gray transparent coating, at first, and later a thick white deposit, sharply contrasting with the dark red border and clean moist tip. (Fischl.)

Vomiting is more frequent in infants, and if associated with constipation suggests a developing meningitis. (Fischl.)

Meteorism in most cases is slight, though in the one just reported it was considerable.

Diarrhea, which is present in about half the cases never begins early, as in this case, but usually sets in late in the disease.

Enlargement of the spleen comes early, and is a very important symptom. Rose spots are present in the great majority of cases, coming on from the 5th to 10th day after the beginning of the fever and appearing in the abdomen and bowels usually. It is strange that in this case neither rose spots were discovered, nor was the spleen palpable at any time during the disease.

Epistaxis is quite rare, but herpes lateralis, which is uncommon in adults is not so in the infantile type.

Urine.—Has the usual febrile characteristic, contains typhoid baccilli in large numbers, and gives a positive Diazo reaction.

Blood changes are very much the same as in adults. There is a moderate decrease in the number of red cells, the hemaglobin is lessened, and there is a slight leucopenia. The most important feature connected with the blood is the Widal reaction, which is positive in from 95 to 98 per cent. of the cases. This is our most important diagnostic procedure. Hemorrhage and perforation is never expected from the pathology.
Relapses occur in 10 per cent. usually red in recovery.

There are fewer nervous symptoms in infantile typhoid, but those which do occur are usually in the second and third weeks, and disappear with the fever. The so-called typhoid state is rare. The little patients are usually dull and apathetic, often in a semi-stupor. Fischl divides the infantile typhoid into three classes, 1. Abortive cases, which are of short duration with either light or severe symptoms. 2. Protracted cases, lasting 5 or 6 weeks. 3. Afebrile cases, frequently overlooked or falsely interpreted.

Relapses occur in 10 per cent., usually bad in recovery.

Complications.—Bronchitis is very frequent and occurs usually toward the end of the first week. Otitis media and pneumonia are fairly common, while meningitis is rather rare. Aphasia has occurred during convalescence, but usually results in recovery.

Diagnosis is usually impossible during the first week. The cause of the temperature, the enlarging spleen and rose spots are the chief diagnostic points, but in many cases the diagnosis must remain in doubt until a positive Widal is obtained toward the end of the first or second week.

It is most likely to be mistaken for malarial colitis, acute miliary tuberculosis, and tuberculous meningitis. From malaria it is distinguished by the difference in the temperature curve, and by the finding of the malarial parasites in the blood. From colitis, by the higher temperature, the palpable spleen and rose spots, the more natural condition of the bowels in typhoid, and by the positive Widal in the latter.

Lumbar puncture and Widal may be necessary to differentiate the disease under discussion from tuberculous meningitis. Prognosis is favorable as a whole. Prophylaxis and treatment are the same in principle as in adults.

30 East Sixty-Second Street.

---

### NEWSPAPER COMMENTS ON SEX HYGIENE.

One of the questions at the present exciting general interest among laymen is that of sex hygiene and the advisability of instructing children and young persons therein. Naturally the discussion rages most fiercely around the question of instruction in sex hygiene in the public schools. Many interesting opinions have been expressed by the newspapers of the country. The judicial thinker must recognize that this question as yet is still in suspense. There is not any unanimity of opinion or agreement on fundamentals sufficient to justify the candid and conservative observer in taking any definite position. Yet the discussion going on through the magazines and newspapers is of the greatest value in bringing to light different sides of the question, and in arousing public interest in the acquisition of more accurate knowledge of these vital subjects.

Stripped of all non-essentials, there are really three questions under discussion. These are:

1. Should children and young persons of preadult ages be instructed in sex hygiene?

2. If so, through what agency should this instruction come? Should it be through one or both parents, through a religious medium, through the public schools, or through books, pamphlets and leaflets of an impersonal character?

3. If it is decided that instruction on this subject should be given in the public schools, what is the best form in which it can be presented to the youthful mind?

Even the most superficial will admit that these three questions present many problems of the utmost complexity. It is difficult to see how any one animated by a calm, judicial spirit can take any other position than that of suspended judgment on the entire question at present. Whatever one may think of the advisability or desirability of sex hygiene nstruction in the abstract, one is reminded of Captain Cuttle's famous saying, "The value of the observation lies in the application of it." The test of any scheme of instruction in sex hygiene is going to lie in the manner in which it is carried out, and in the tact, good judgment and common sense of the persons doing the teaching, whether they be parents, religious teachers, school-teachers or writers.

The reflection of opinions on the part of the newspaper editors is interesting as showing the diversity of views expressed.

The Seattle (Wash.) Post-Intelligencer, commenting on the objections of the local superintendent of schools, says, "That sex hygiene is a matter for the future is too probable to admit of much doubt, but just now such instruction is decidedly impracticable . . . Before any such revolutionary change in the general notions of sex matters as is implied in the teaching of sex hygiene can be made, the vast body of public opinion must be attuned to it and made to recognize its necessity, not through controversy, but through intelligent appreciation of certain facts. To this end the present discussion for and against eugenics, foolish as it often is on both sides of the argument, is working. That the fundamental physical problems of life, once rigorously tabood, are now permissable topics for conversation is a sure index of the direction in which we are going."

The Post-Intelligencer takes a sound view of the value of present discussion, without in any way yielding its judgment as to its ultimate opinion. It is also perfectly sound in holding that the question "will solve itself gradually and quietly within the next generation, and the desired end will be attained at far less cost and with far more speed if it is not hampered with overanxiety for immediate results."

The Waterloo (Iowa) Courier, starting out with the proposition that "health is the foundation on which rests the happiness of a people and the power of a nation," lays particular stress on the necessity of public-school education in hygiene and sanitation. After elaborating an extensive program for public-school education, it says, "The health movement in our public schools has been transformed during the past decade from a purely negative movement, having as an object the avoidance of disease, to a splendidly positive movement, having for its aim the development of vitality." While the subject of sex hygiene is not specifically included in the Courier program, it evidently assumes that this will follow. "In these schools the physical, the mental and the moral will be developed together and not separately."

The Beloit (Wis.) News states that "wide difference of opinion still exists among schoolmen on this subject, although they are united in recognizing what President Foster of Reed College calls 'the social emergency,' the separation coming when the details of the exact part the public schools play in this campaign are taken up." After discussing the opinions of various educators, the News concludes that this question is assuming increasing importance in our school system, and that some practical solution for the problems involved will have to be devised.

The Fort Worth (Texas) Record "seriously doubts the wisdom of any attempt of education in the public schools on this delicate question." It regards the subject as one of such sacredness that it should not be allowed to become a topic of careless and common conversation among the bold and irreverent Young Americas that constitute the school population. In the opinion of the Record, the proper teachers on such subjects are the fathers, mothers and family physicians. "The modern tendency to put all responsibilities of instruction on schoolteachers is dangerous in the extreme . . . The public schools cannot take the place of the home or the church." While the Record recognizes the fact that "there are children without intelligent or watchful parents and without religious surroundings, and that these exceptions deserve consideration," it is of the opinion that a great mass of children in the United States come from homes and have no need of tuition in morals in the public schools. "Let the home, the physician and the church bear their own responsibilities in rearing the rising generation," is its final conclusion.

The Chicago Record-Herald, discussing the bulletin of the federal Bureau of Education on this subject, and the conflicting views expressed therein, finds nothing strange or unusual in this divergence of opinion. The learned doctors never agree, and if society waited for unanimous consent of the experts nothing would ever be done. Questions are settled by experience, not by disputation." The Record-Herald's views on the outcome of reform movements and controversies are especially interesting. "Questions are forgotten, not settled. Unconvinced parties give up and take up new issues." The editor's application of this philosophy to the problem of sex hygiene is that sex hygiene as a course of study is undoubtedly coming in this country. The really significant fact is that so many educators, social workers, physicians and moralists have been converted to the idea in such a short space of time. The results it is willing to leave to the future.

The Wilkes-Barre (Penn.) Record asks, "Should sexology be introduced into the schools as a study or by way of a series of lectures?" After stating the arguments on both sides, the Record concludes, "We believe the weight of the argument is in favor of those who advocate a simple form in instruction for children who have reached the last year or two of grammar school." Believing that the amazing amount of evil from vice is the result of ignorance, the Record favors instruction which would "follow up the laws of Nature as revealed in plant life and animal life, so as to be without the element of harmful suggestives." Drawing a comparison between the evils done by intemperance and those caused by sexual vices, the editor concludes, "More can be done in the way of abstention from alcoholic indulgence by impressing young people with the astonishing physical consequences than in any other way, and so it is with the almost incredible consequences of sexual dissipation."

Probably no newspaper in New England is more ably edited or exercises a wider influence than the Boston Transcript. Its editoral point of view is sane and conservative. Its opinion is that "altogether educators and physicians have not yet agreed as to whether sex hygiene should be taught in the public schools, or how it should be taught if at all, it must be admitted that the whole question of sex education is assuming great importance in our city school systems."

The Philadelphia Ledger, on the other hand, regards the present agitation as a passing fad. "The zeal of the advocates of the proposed change is analogous to the zeal of the ultra-progressives who would reform the world by legislation." The Ledger's criticism of advocates of radical legislation for which no public support has been developed is thoroughly sound. "To such persons, an act placed on the statute books seems the end of positive effort for the betterment of the world. This secured, vigilance subsides and the honest-motived but illdirected reformer settles down to a complacent satisfaction." While this is true of many reformers and agitators who seek to accomplish moral ends by legislation, it can hardly be applied with fairness to those advocating education and instruction as a means of influencing action. The Ledger's question, "Why should any adult impose his alarmist instruction on the youth?" is easily answered. It is because the adult has seen enough of the results of youthful folly to justify his alarm and to lead him to guard the youth from repeating and perpetuating the mistakes of previous generations. This is the only reason why adults are teachers and why youths are pupils. Age has accumulated knowledge and experience which it would hand down to youth. Summing up its argument, the Ledger decides that public schools do not need a course of sex hygiene. "They may need a healthier-minded attitude on the part of all teachers of biology, hygiene and physical instruction." In the opinion of the Ledger, the teachers opposing sex hygiene instruction have a more correct attitude of mind toward their subject than have the advocates of the plan, which the Ledger regards as a course "full of peril and demoralizing possibilities."

In addition to its editorial, the Ledger gives space to a number of letters discussing both sides of the question. One correspondent, while admitting that "the proposed new teaching is not without danger," hopes that eventually the letting in of light on this subject "will lead up to that happy condition when there will be no more nasty prudery about describing sex to mixed classes than there is now in describing the stamens and pistils of flowers." The correspondent rather cleverly attempts to spike the guns of the Ledger's editorial by quoting from an editorial which appeared in the next column on "Degeneracy in a Little Town," in which the repulsive conditions found in a Newfoundland village are described, with the editorial comment that "the difficulty seems to be that these people are content to be no better than their fathers were before them." While admiring the correspondent's ingenuity, it must be admitted that he is begging the question, since the point which the Ledger is discussing is whether such a course would make the children better than their fathers. Another correspondent takes the opposite side of the question, quoting Plato, Emerson, Rousseau and Herbert Spencer in support of his proposition that "the teaching of this delicate subject is for the parents, the church and the doctor."

The Chicago Tribune sums up the question in this fashion: "It is not a question of whether the youth shall remain ignorant or be instructed. It is a question whether it shall be well or ill instructed."

Collier's Weekly holds that "the argument for teaching sex hygiene is being cried in agonies. . . . The sob of a mother whose baby must go through life blind because of some one's ignorance is an argument for sex education that defies the glibbest debator."

Especially important as showing the probable attitude of the Roman Catholic Church on this question is an editorial in America, which designates the proposition to teach sex hygiene in the public schools as "the newest and most dangerous of all the educational fads now being daily foisted on us." The argument of America is for development of character in the public schools, rather than for an increase in detailed instructions in facts. "It is not the law of the mind that needs strengthening, but the law of the members that needs restraining; not more knowledge but more will is required." One kind of instruction in sex hygiene would meet the approval of America. "Instead of being worried about increasing the knowledge, be energetic and persistent in decreasing the desire. . . . It is the incentive that should be removed." The way to better conditions lies, in the judgment of America, in improving social conditions rather than in specific instruction of schoolchildren. "Let the sex hygienists put away the countless seductions which assail mankind and womankind on all sides, and they will effect something. Let the young have less desire, not more knowledge; strength of will, not complete information."

While recognizing the necessity of developing personal character and will power, as well as the uselessness of increasing knowledge without developing personal qualifications to correspond, America's argument is weak, in that it objects to a specific method of instruction and offers instead a vague general plan of reform. Doubtless, the sex hygienist would be glad to "put away the countless seductions which assail mankind and womankind on all sides." But how is such a stupendous task to be accomplished? To object to proper instruction in the public schools, and to offer as a substitute a reform of newspapers, theaters, fashions, current fiction and habits of living hardly seems to be a practical criticism.

The Los Angeles Graphic thinks that such instruction is a duty to parents, but that 90 per cent, of American parents "dodge the responsibility of instructing their children properly in sex matters."

A correspondent of the New York Independent denounces the idea as "the most vicious of all the wild, indiscreet and dangerous suggestions made in behalf of the human race." The Independent correspondent is a true optimist. He says, "The world is growing better all the time and will continue if let alone on such subjects as this." If this is true, the pathway to the millennium is a straight and easy one.

In a multitude of counselors there may be safety; but with the safety is combined great diversity of opinion. What conclusion can be drawn from this discussion, which has evidently only just begun? None, at present, except that the need of adopting some plan by which the disastrous effect of social evils can be diminished is apparently admitted by all. The discussion then turns on the important questions of when, where and how. Where shall the child be instructed: at home, in the church on in the school? If the Los Angeles Graphic is correct and 90 per cent. of American parents are dodging their duty, then one of two courses must be adopted. Either parents must be educated to a realization of their responsibility, or some other agency must take the place of the parent in education on this subject. If a substitue for the parent must be found, which shall it be, the church or the public school? If the church, to-day, using this term in its broadest and most comprehensive sense, were attracting or holding the greater part of our population, and if a majority of the children of public-school age were coming under the influence of the church, this might be considered as an ideal arrangement. But what are the facts? We have in most states compulsory school laws. We have not nor can we have any compulsory church laws. Only those who desire church relations come under religious influences. The most liberal estimate, accepting the figures of the churches themselves, show that only 35,000,000 out of our population of about a hunderd million are directly connected with any religious organization of any character. The church as an educational factor in this field, therefore, would have an efficiency of only 35 per cent. If the church will undertake this responsibility for those children whom it can reach, by all means let them do so. But what of the 65 per cent. outside its influence?

So much for the answer to the first question. As to the second and third questions, when and how, these must be settled by our authorities in psychology and practical pedagogy. Public interest in the subject is unquestoned. To repeat, it is not a matter to be settled by heated controversy but by calm and judicious consideration. Above all, it is not a matter to be controlled by the "fringe of fanatics" which as Theodore Roosevelt has aptly said, "hang on the outskirts of every reform movement." The question is not as yet one for dogmatic assertion but for careful consideration and suspended judgment.

Jour. A. M. A. Oct. 18, 1913.

